# MRI‐guided interventional natural killer cell delivery for liver tumor treatment

**DOI:** 10.1002/cam4.1459

**Published:** 2018-03-30

**Authors:** Zhanliang Su, Xifu Wang, Linfeng Zheng, Tianchu Lyu, Matteo Figini, Bin Wang, Daniel Procissi, Junjie Shangguan, Chong Sun, Liang Pan, Lei Qin, Bin Zhang, Yury Velichko, Riad Salem, Vahid Yaghmai, Andrew C. Larson, Zhuoli Zhang

**Affiliations:** ^1^ Department of Radiology Feinberg School of Medicine Northwestern University Chicago Illinois 60611; ^2^ Hematology/Oncology Northwestern University Chicago Illinois 60611; ^3^ Robert H. Lurie Comprehensive Cancer Center Chicago Illinois 60611

**Keywords:** Adoptive transfer immunotherapy, hepatocellular carcinoma, interventional oncology, magnetic resonance imaging, natural killer cell

## Abstract

While natural killer (NK) cell‐based adoptive transfer immunotherapy (ATI) provides only modest clinical success in cancer patients. This study was hypothesized that MRI‐guided transcatheter intra‐hepatic arterial (IHA) infusion permits local delivery to liver tumors to improve outcomes during NK‐based ATI in a rat model of hepatocellular carcinoma (HCC). Mouse NK cells were labeled with clinically applicable iron nanocomplexes. Twenty rat HCC models were assigned to three groups: transcatheter IHA saline infusion as the control group, transcatheter IHA NK infusion group, and intravenous (IV) NK infusion group. MRI studies were performed at baseline and at 24 h, 48 h, and 8 days postinfusion. There was a significant difference in tumor R2* values between baseline and 24 h following the selective transcatheter IHA NK delivery to the tumors (*P *=* *0.039) when compared to IV NK infusion (*P *=* *0.803). At 8 days postinfusion, there were significant differences in tumor volumes between the control, IV, and IHA NK infusion groups (control vs. IV,* P *=* *0.196; control vs. IHA,* P *<* *0.001; and IV vs. IHA,* P *=* *0.001). Moreover, there was a strong correlation between tumor R2* value change (∆R2*) at 24 h postinfusion and tumor volume change (∆volume) at 8 days in IHA group (*R* ^2^ = 0.704, *P *<* *0.001). Clinically applicable labeled NK cells with 12‐h labeling time can be tracked by MRI. Transcatheter IHA infusion improves NK cell homing efficacy and immunotherapeutic efficiency. The change in tumor R2* value 24 h postinfusion is an important early biomarker for prediction of longitudinal response.

## Introduction

Hepatocellular carcinoma (HCC) is the fifth most common malignancy in the world and the fourth leading cause of cancer death in the United States 1, 2. Surgical resection and transplantation are potentially curative treatments for HCC, but only 10–15% of patients are candidates 1, 2. The overall survival (OS) benefits from current liver‐directed transcatheter approaches remain relatively modest 2, 3, 4.

While natural killer (NK) cell‐based adoptive transfer immunotherapy (ATI) holds great promise for clinical cancer treatment, only modest clinical success has been achieved thus far using NK‐ATI therapies in cancer patients 5, 6. Two challenges hurdle for NK‐ATI in HCC patients include: (1) inadequate homing efficiency of NKs to the targeted tumors and (2) lack of well‐established noninvasive tools for predicting the NK‐ATI response 7, 8, 9.

We used a clinically applicable approach combining the FDA‐approved drugs *h*eparin, *p*rotamine, and *f*erumoxytol to form *HPF* nanocomplexes for magnetic NK labeling so that NK cell biodistribution could be visualized in vivo with advanced magnetic resonance imaging (MRI) following transcatheter intrahepatic arterial (IHA) local delivery.

The purposes of our study was to test the hypotheses in a rat model of hepatocellular carcinoma: (1) clinically applicable labeled NK cells can be tracked with MRI, (2) transcatheter IHA NK infusion improves NK cell homing efficacy to targeted tumors, and (3) serial MRI monitoring of NK cell migration to targeted tumors can serve as an early biomarker for prediction of longitudinal response.

## Materials and Methods

All studies were approved by our institutional animal care and use committee and were performed in accordance with the NIH guidelines.

### Cell lines and culture

Mouse NK cell line (LNK) 10 was kindly obtained by Stephen K. Anderson (National Cancer Institute, Frederick, MD). The LNK cell line was cultured in RPMI 1640 containing 10% fetal bovine serum (FBS), 100 U/mL penicillin, 100 U/mL streptomycin, 1.5 g/L sodium pyruvate, l‐glutamine, and IL‐2 (8000 IU/mL) at 37°C in a humidified atmosphere with 5% CO_2_ air. McA‐RH7777 (McA) is buffalo rat hepatoma cell line (ATCC, CRL‐1601, Manassas, VA) 11 was cultured in Dulbecco's modified Eagle's medium (DMEM, ATCC, Manassas, VA) containing 10% FBS, 100 U/mL penicillin, and 100 U/mL streptomycin at 37°C with 5% CO_2_ air. Trypan blue staining was performed before each tumor implantation procedure to verify >90% cell viability.

### HPF nanocomplex preparation

FDA‐approved ferumoxytol (Feraheme^®^, AMAG Pharmaceuticals, Inc., Boston, MA) is an ultra‐small superparamagnetic iron oxide nanoparticle with a size of 17–31 nm in diameter for treatment of iron deficiency anemia. FDA‐approved heparin sulfate and protamine sulfate (both from American Pharmaceuticals Partner, IL) were used to form HPF nanocomplexes 12. The HPF complexes were prepared by sequentially adding heparin at 2 U/mL and protamine at 60 *μ*g/mL with ferumoxytol at 50, 100, or 200 *μ*g/mL, separately 12, 13 (L.Z., B.W., and Z.Z).

### HPF nanocomplex labeling of LNKs

HPF nanocomplexes (with 0, 50, 100, or 200 *μ*g/mL ferumoxytol) in 0.5 mL phosphate‐buffered saline (PBS) were added to each flask (5 × 10^6^ LNKs in 10 mL culture medium), separately. Each flask was then incubated for 12 h. Labeled cells were harvested and washed with PBS and then with 10 U/mL heparin to remove the residual HPF 12. Cells were then fixed for Prussian blue (Invitrogen, Carlsbad, CA) staining and transmission electronic microscopy (TEM) (FEI Company, Hillsboro, OR). Cell labeling efficiency (%) = number of positive‐staining cells/number of total cells in per ten fields (20×) (S.Z. and Z.Z.). TEM images of LNKs were used to verify intracellular iron uptake. A separate set of samples was used to quantify iron content within each cell using inductively coupled plasma mass spectrometry (ICP‐MS, Thermo Fisher Scientific Inc., Waltham, MA), as previously described 14 (L.Z. and Z.Z.).

### Labeled NK cell viability and cytotoxicity function assays

The cell viability assay methods were described in previous studies 14, 15. Aliquots with 5 × 10^5^ cells each were resuspended in 100 *μ*L annexin V binding buffer (BD Pharmingen, San Diego, CA) and incubated with 5 *μ*L FITC‐conjugated annexin V reagent (BD Pharmingen), either with or without 10 *μ*L of 50 *μ*g/mL propidium iodide solution (PI, BD Pharmingen) for 15 min in the dark. These cells were then analyzed using a FACScalibur flow cytometer (BD Biosciences, San Jose, CA), yielding percentages of apoptotic (annexin V positive, PI negative), necrotic, and viable cells. The study was repeated with 0, 50, 100, or 200 *μ*g/mL ferumoxytol labeling protocol, and cell viabilities were assessed at 3 days after the 12 h labeling period (each group, *N *=* *6).

NK cells were labeled with HPF nanocomplexes (50 *μ*g HPF/mL with 10^6^ NK cells for 12 h labeling). McA target cells were labeled with carboxyfluorescein succinimidyl ester (CFSE, Sigma‐Aldrich, St. Louis, MO) to discriminate target cells from effector cells. Labeled and unlabeled or HPF‐labeled NK effector cells were co‐cultured with CFSE labeled McA target cells at different effectors to target (E/T) ratios at 25:1, 12.5:1, and 6.2:1 in 96‐well U‐bottomed microplates, separately. Additionally, two sets of controls containing only effector cells or only target cells were prepared. After overnight co‐culture, the cells were harvested and stained with 7‐AAD (BD Pharmingen) and pacific blue Annexin V for 15 min in the dark. These cells were then analyzed using a FACScalibur flow cytometer. All conditions were performed five times independently. NK cytotoxicity (%) is calculated as the number of cells positive for both CFSE and Annexin V divided by the total number of CFSE positive cells, after subtracting the spontaneous apoptosis (%) in target cell only controls (B.W., B.Z., Z.Z., and L.Q.) 16.

### Animal model

Twenty male buffalo rats (body weight 250–300 g, Charles River Laboratories, Wilmington, MA) were anesthetized. A mini‐laparotomy was performed, and 2 × 10^6^ McA cells in 0.2 mL PBS were injected in the left median and left lateral lobes. Tumors were allowed to grow for 7 days to reach a size typically ≥5 mm in diameter verified by MRI. These rats were separated into three groups: transcatheter IHA saline infusion as the control group, transcatheter IHA LNK infusion group, and intravenous (IV) LNK infusion group (each group *N *=* *6, 12 tumors, a total of 36 tumors included) (Z.S., T.L., X.W., M.F., and Z.Z.) 17, 18.

### Transcatheter IHA LNK local infusion

Transcatheter IHA infusion methods were described in previous studies 17, 18, 19. In brief, the rat was anesthetized, and a mini‐laparotomy was performed. The common hepatic artery was clamped to temporarily prevent bleeding, and a 4‐0 suture was used to permanently ligate the gastroduodenal artery to prevent backwards flow. A 24‐gauge microcatheter was placed in common branch of the proper hepatic artery with placement confirmed by digital subtraction angiography (DSA) guidance (Omnipaque, GE Healthcare, Pittsburgh, PA). Catheter was placed in the left hepatic artery, and 0.1 mL of heparin was infused before infusing 4.0 × 10^6^ labeled LNKs (50 *μ*g/mL HPF labeling 12 h) suspended in 0.3 mL PBS. After LNK infusion, abdominal incisions were closed. For the transcatheter IHA saline infusion control group, 0.5 mL of PBS was infused, comparable in volume to the LNK suspension. All other procedures were done by the exact same method as transcatheter IHA LNK infusion group (Z.S., T.L., X.W., M.F., and Z.Z.).

### LNK systemic delivery

4 × 10^6^ labeled LNKs (50 *μ*g/mL HPF labeling 12 h) in 0.5 mL of PBS were infused via tail vein followed by 0.2 mL of saline flush and 0.1 mL of heparin lock flush.

### MRI of cell phantoms

MRI was performed using a 7.0 T 30 cm bore scanner (ClinScan, Bruker BioSpin, Ettlingen, Germany) with 75 mm rat coil (Bruker BioSpin). MRI sequences and parameters are listed in Table 1. Labeled cells using 50 *μ*g/mL HPF labeling for 12 h were fixed with 10% formalin. LNK cell phantoms contained 1 × 10^6^ and 2 × 10^6^ labeled LNKs, 2 × 10^6^ unlabeled LNKs, or only 1% agarose. All cells were homogenously suspended in 1 mL 1% agarose gel (each *N *=* *6). These mixtures were then transferred to 1.5 mL tubes for MR phantom studies (D.P., L.Z., and Z.Z.).

**Table 1:** MRI sequences and parameters

Sequence	Cell phantom		In vivo MRI		
	GRE T2*W	GRE T2* map	TSE anatomy	GRE T2 map	GRE T2* map
TR/TE (msec)	50/10.00	1100/(6.00, 12.18, 21.45, 66.18, 72.36)	T1W: 200/2.6 T2W: 841/28.00	1300/(9.3, 18.6, 27.9, 37.2)	400/(3.28, 6.14, 9.00, 11.86)
FA	9°	30°	T1W/T2W: 90°/180°	180°	90°
FOV (mm^2^)	45 × 45	57 × 57	60 × 60	60 × 60	60 × 60
Matrix	128 × 128	128 × 128	192 × 192	192 × 192	192 × 192
ST (mm)	0.30	1.5	1.0	1.0	1.0
VS (mm^3^)	0.35 × 0.35 × 0.30	0.45 × 0.45 × 1.50	0.31 × 0.31 × 1.00	0.31 × 0.31 × 1.00	0.31 × 0.31 × 1.00

FA, flip angle; FOV, field of view; GRE, Gradient echo sequences; TE, echo time; TR, repetition time; TSE, turbo spin echo; ST, slice thickness; VS: voxel size.

### In vivo MRI tracking of labeled LNK cell biodistribution in rat liver

MRI scans were performed before (baseline) and at 24 h, 48 h, and 8 days after injection using the 7.0 T scanner with 75 mm rat coil. T2 and T2*/R2* mapping was performed following acquisition of TSE T1‐weighted (T1W) and T2W anatomical images. Scan parameters are listed in Table 1. Mean R2* (1/T2*) values for the tumors were measured pre‐ and postinfusion of LNKs (24 h and 48 h) (M.F., X.W., and S.Z.).

### Assessment of therapeutic response using T2W images

The volume of each tumor was measured on axial T2W images as previously described 20. These measurements were performed for both baseline images and images collected at 8 days postinfusion. The change of tumor volume (Δvolume) was calculated using the formula: Δvolume = volume 8 days − volume at baseline (Z.S. and X.W.).

### Histology

After completion of MRI 8 days postinfusion, all rats were euthanized. Livers were harvested and fixed in 10% formalin, and then, tissues were embedded in paraffin. Sections including tumor tissues were sliced (4 *μ*m) for CD56 (Anti‐CD56, Becton Dickinson, San Jose, CA) immunohistochemistry (IHC) staining 17. Resulting histology slides were reviewed by a pathologist at Pathology Core Facility (L.L., MD, who had more than 10 years of experience).

### Image analysis

For MRI examinations, image analyses were performed using MATLAB (2011a, MathWorks, Natick, MA). T2*/R2* maps were generated. Using both the T2W MR Images as anatomical reference to identify the tumor boundaries, region of interest (ROI) was drawn to completely cover the viable tumors to measure R2* values; four slices were included for each tumor for R2* measurements. ∆R2* values were calculated (∆R2* = tumor R2* at 24 h postinfusion − tumor R2* at baseline) (X.W. and Z.S.). CD56 stained slides (4 slices/each tumor) were scanned at a magnification of 20× and digitized using the TissueFAXS system (TissueGnostics, Los Angeles, CA). These acquired images were analyzed using the HistoQuest Cell Analysis Software (TissueGnostics) package to quantify the total number of LNK cells within each specimen (T.L., X.W., and Z.S.).

### Statistical analysis

Data are presented as mean ± standard deviation (SD) as indicated. One‐way ANOVA was used to compare in vivo R2* and tumor volume among animal groups (control, IHA, and IV) at baseline. *t*‐tests were used to compare R2* and tumor volume of different groups measured at different time‐points (baseline, 24 h, 48 h, and 8 days after infusion). Changes of R2* (ΔR2*) and tumor volume (Δvolume) were also compared among groups at different time points using *t*‐test. Pearson's correlation coefficient (*r*) was also applied to assess the relationship between ΔR2* measurements and Δvolume measurements. Statistical analysis was performed using SAS 9.4 (SAS Institute, Inc., Cary, NC) by a biostatistician (K.L.).

## Results

### NK cell labeling efficiency and iron content in each cell

**Figure 1:**
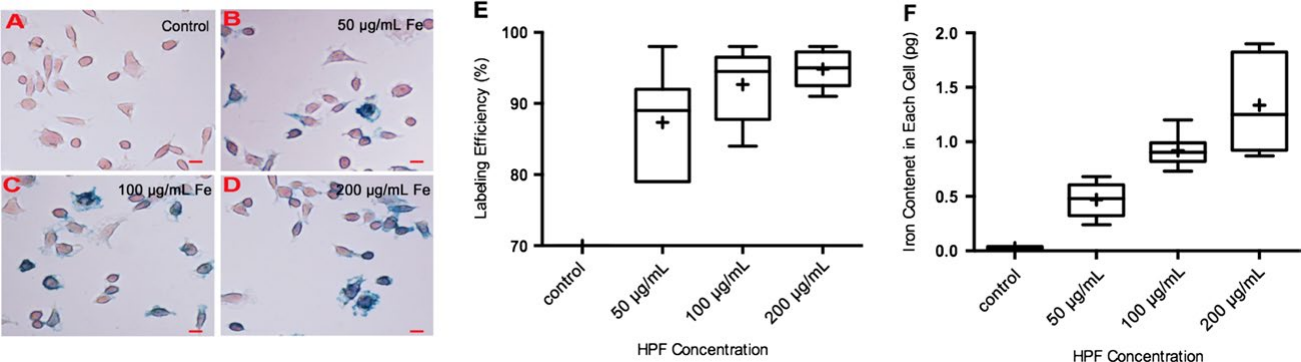
Prussian blue staining of LNKs with 12‐h labeling period, cell labeling efficiency, and iron content in each cell. A–D, representative Prussian blue‐stained LNKs. A, unlabeled LNKs; B, labeled LNKs with 50 *μ*g/mL HPF; C, labeled LNKs with 100 *μ*g/mL HPF; and D, labeled LNKs with 200 *μ*g/mL HPF. E, Cellular uptake efficiency increased with exposure to a higher concentration of HPF during labeling procedures (50 µg/mL HPF group vs. 100 µg/mL HPF group, *p* = 0.1783; 50 µg/mL HPF group vs. 200 µg/mL HPF group, *p* = 0.0535; 100 µg/mL HPF group vs. 200 µg/mL HPF group, *p* = 0.3916). F, cellular iron concentration (pg/cell) was measured by ICP‐MS (each *N *=* *6) (control vs. 50 *μ*g/mL,* P *<* *0.001; control vs. 100 *μ*g/mL,* P *<* *0.001; control vs. 200 *μ*g/mL,* P *<* *0.001; 50 *μ*g/mL vs. 100 *μ*g/mL,* P *=* *0.178; 50 *μ*g/mL vs. 200 *μ*g/mL,* P *=* *0.054; 100 *μ*g/mL vs. 200 *μ*g/mL,* P *=* *0.392). A–D scale bars = 10 *μ*m.

**Figure 2:**
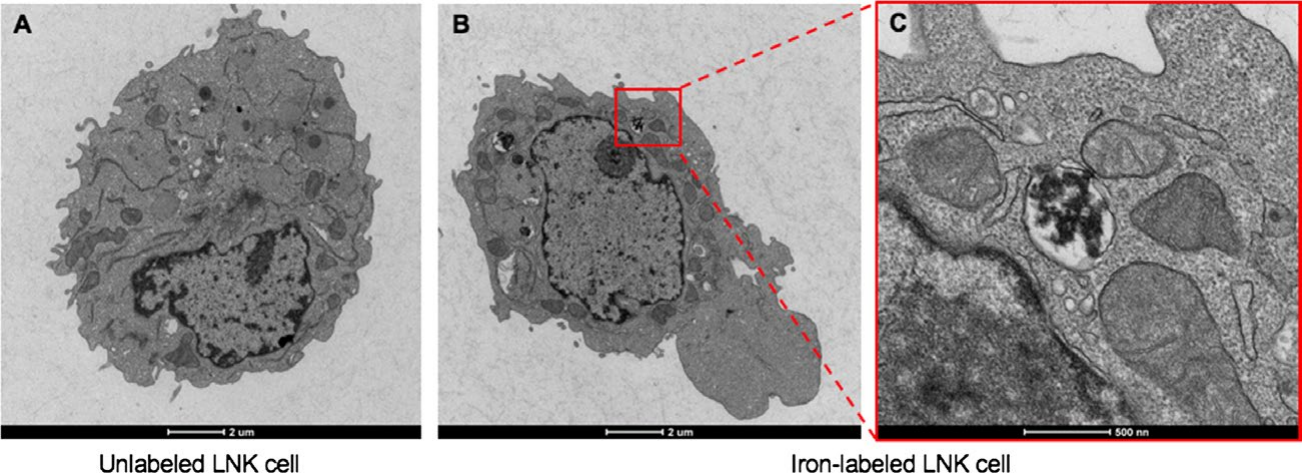
Representative TEM images. A, TEM images of a single unlabeled LNK; B, a single HPF‐labeled LNK using 50 *μ*g/mL HPF with 12‐h labeling period. TEM images demonstrated the localization of iron oxide particles within the cytoplasm and cytoplasmic vesicles of LNK cell (B), and C a magnified view of the square area in B.

Microscopic observation of Prussian blue‐stained LNKs revealed extensive, nonuniform uptake of iron particles (Fig. 1A–D). The iron content of the unlabeled cells was significantly lower than that of the labeled cell groups (Fig. 1E). Labeling efficiency measurements using Prussian blue assays are 0% for control with HPF, 87 ± 7% for 50 *μ*g/mL HPF group, 92 ± 5% for 100 *μ*g/mL HPF group, and 95 ± 11% for 200 *μ*g/mL HPF group. Uptake of iron nanoparicles was confirmed in labeled LNKs, but was not observed in unlabeled LNKs by TEM (Fig. 2A). The internalization of iron nanoparticles in the cytoplasm was confirmed and was not observed in the LNK cell membrane (Fig. 1B–C) with 0, 50, 100, or 200 *μ*g/mL.

### NK cell viability and cytotoxicity function

**Figure 3:**
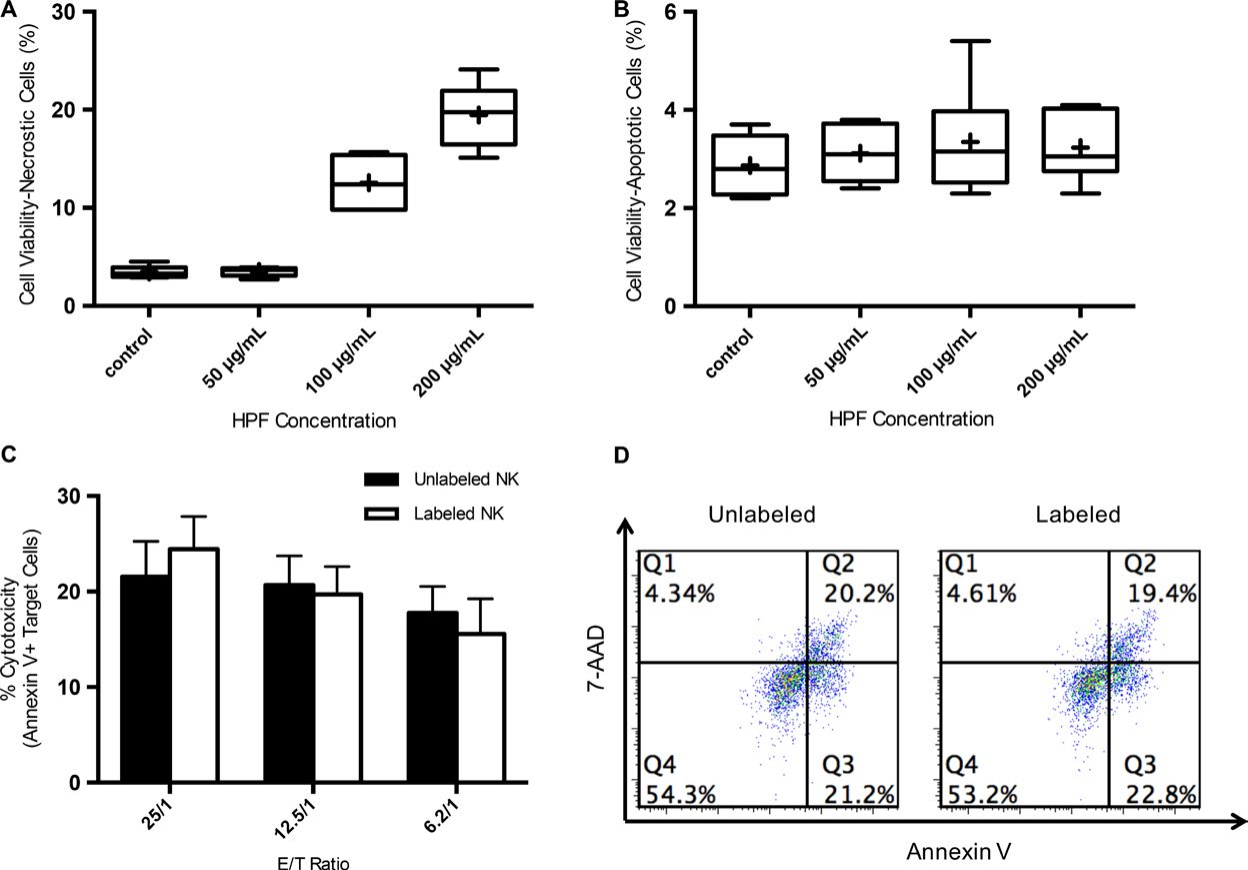
LNK cell viability and assessments at day 3 after the 12‐h labeling period and cytotoxicity assessments (each *N *=* *6). A, a significant increase in the percentage of cell death was found for 100 *μ*g/mL and 200 *μ*g/mL HPF labeled groups compared to control group (100 *μ*g/mL vs. control, *P *<* *0.001; 200 *μ*g/mL vs. control, *P *=* *0.002); however, there was no significance between 50 *μ*g/mL group and control group (*P *=* *0.875). B, the percentage of apoptotic cells were measured by FACS, and there were no significant differences between unlabeled group and labeled groups (control vs. 50 *μ*g/mL,* P *=* *0.493; control vs. 100 *μ*g/mL,* P* = 0.374; control vs. 200 *μ*g/mL,* P *=* *0.36; 50 *μ*g/mL vs. 100 *μ*g/mL,* P *=* *0.659; 50 *μ*g/mL vs. 200 *μ*g/mL,* P *=* *0.762; 100 *μ*g/mL vs. 200 *μ*g/mL,* P *=* *0.831). C, NK cell killing of McA cells at three different effector to target ratios (E/T) (Group 25/1: labeled and unlabeled, *P *=* *0.19; Group 12.5/1, *P *=* *0.579; Group 6.2/1, *P* = 0.267). D, representative flow dot plots of (C) at 25:1 (E/T).

At 3 days after the 12‐h labeling period, there were no significant differences in the percentages of apoptotic cells in all groups, while significant increases in percentages of necrotic cells were observed in 100 *μ*g/mL and 200 *μ*g/mL compared to control group F (Fig. 3A and B). For cytotoxicity function assay, there was no significant difference between labeled and unlabeled NK cells at all target effector ratios (Fig. 3C). The percentage of early apoptosis and late apoptosis of target cells caused by unlabeled and labeled NK cells also showed no significant difference at all ratios, and representative flow dot plots of (Fig. 3C) at 25:1 (E/T) are shown in Figure 3D.

### In vitro MRI of phantoms

**Figure 4:**
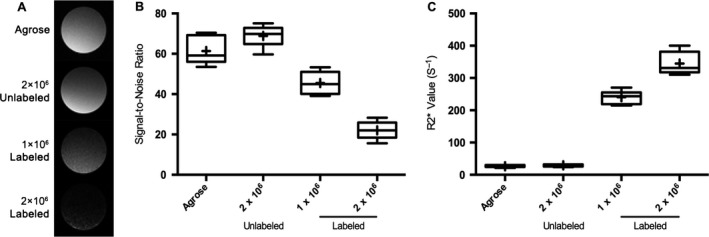
Qualitative MRI of cell phantoms (each *N *=* *6). A, T2* signal intensity of the NK cell phantoms containing different numbers NKs labeled with 50 *μ*g/mL HPF. B, SNR values decreased in proportion to the number of labeled NKs included in cell phantoms (agarose vs. 2‐million unlabeled cells, *P *=* *0.062; agarose vs. 1‐million labeled cells, *P *=* *0.001, agarose vs. 2‐million labeled cells *P *<* *0.001; 2‐million unlabeled vs. 1‐million labeled cells, *P *<* *0.001; 2‐million unlabeled vs. 2‐million labeled cells, *P *<* *0.001; 1‐million labeled vs. 2‐million labeled cells, *P* < 0.001). C, R2* values increased in proportion to the number of labeled NKs included in cell phantoms (agarose vs. 2‐million unlabeled cells, *P *=* *0.19; agarose vs. 1‐million labeled cells, *P *<* *0.001, Agarose vs. 2‐million labeled cells, *P *<* *0.001; 2‐million unlabeled vs. 1‐million labeled cells, *P *<* *0.001; 2‐million unlabeled vs. 2‐million labeled cells, *P *<* *0.001; 1‐million labeled vs. 2‐million labeled cells, *P *<* *0.001). SNR represents signal‐to‐noise ratio.

The T2* signal‐to‐noise ratio (SNR) of the LNK cell phantoms containing different numbers of LNKs labeled with 50 *μ*g/mL HPF markedly decreased with increasing cell number (Fig. 4A). SNR values decreased in proportion to the number of labeled LNKs included in each cell phantom in Figure 4B. R2* values increased in proportion to the number of labeled LNKs included in each cell phantom in Figure 4C.

### In vivo quantitative MRI measurements

One rat displayed no growth (no tumor detected) and thus was excluded from the study. Another rat was excluded because of poor health at baseline imaging time‐point.

**Figure 5:**
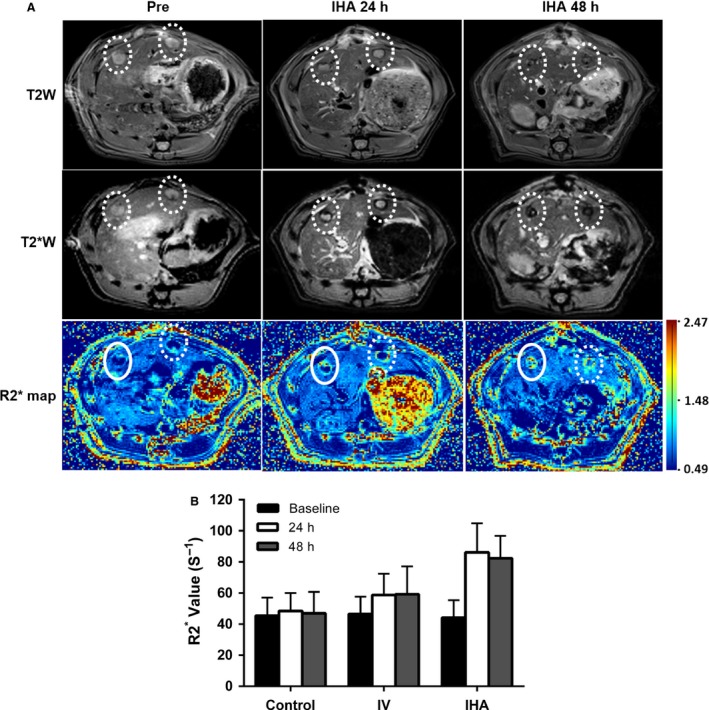
Quantitative MRI of LNK cell biodistribution in the targeted tumors. A, representative T2W and T2*W images and R2* maps at pre‐ and postinfusion intervals (24 h and 48 h). T2W images at preinfusion, postinfusion 24 h and 48 h are on the top row. T2*W images at preinfusion, postinfusion is on the middle row. Quantitative R2*maps at preinfusion, postinfusion 24 h and 48 h are on the bottom row. B, tumor R2* values at pre‐ and postinfusion intervals (each group *N *=* *12) (IHA group: baseline vs. 24 h postinfusion, *P *=* *0.039; baseline vs. 48 h postinfusion, *P *=* *0.019; 24 h vs. 48 h postinfusion, *P *=* *0.754, control group, baseline vs. 24 h postinfusion, *P *=* *0.741; baseline vs. 48 h postinfusion, *P *=* *0.592; 24 h vs. 48 h postinfusion, *P *=* *0.568. IV group, baseline vs. 24 h postinfusion, *P *=* *0.803; baseline vs. 48 h postinfusion, *P *=* *0.694; 24 h vs. 48 h postinfusion, *P *=* *0.907). Tumor R2* value at baseline: control vs. IV,* P *=* *0.877; control vs. IHA,* P *=* *0.991; IV vs. IHA,* P *=* *0.9. Tumor R2* value at 24 h postinfusion, control vs. IV,* P *=* *0.676; control vs. IHA,* P *=* *0.041; IV vs. IHA,* P *=* *0.023. Tumor R2* value at 48 h postinfusion, control vs. IV,* P *=* *0.719; control vs. IHA,* P *=* *0.006; IV vs. IHA,* P *=* *0.006. Circles indicate tumor locations.

Following tumor implantation at 7 days, tumor volume was measured in T2W images. The mean baseline tumor volumes for control, IV, and IHA groups were 65.69 ± 18.10 mm^3^, 64.35 ± 18.26 mm^3^, and 64.07 ± 11.53 mm^3^, respectively. The tumors were depicted with relatively homogeneous high signal intensity before the delivery of labeled NKs in baseline T2W and T2*W images. Representative axial T2W images, T2*W images, and R2* maps from one of the IHA group animals were shown (Fig. 5A).

Identification of the IHA and its subsequent catheterization was confirmed by DSA, and the success rate was 100%. For IHA LNK group, both 24 h and 48 h postinfusion signal intensities within the tumor nodules became heterogeneous. Figure 5B shows: (1) There were no significant differences in baseline tumor R2* values of the three groups. A significant difference in R2* values was found between baseline and 24/48 h after IHA LNK cell infusion within tumor tissues, but there was no significant difference in tumor R2* values between the 24 h and 48 h follow‐up period and (2) there were no significant differences in R2* values between baseline and 24/48 h; between 24 h and 48 h after IV LNK cell infusion, but increasing R2* value trends were observed between baseline and 24/48 h. However, there were significant differences in tumor R2* values between control, IV, and IHA groups at both 24 h and 48 h postinfusion time‐points (Fig. 5B).

### Therapy response and histology

**Figure 6:**
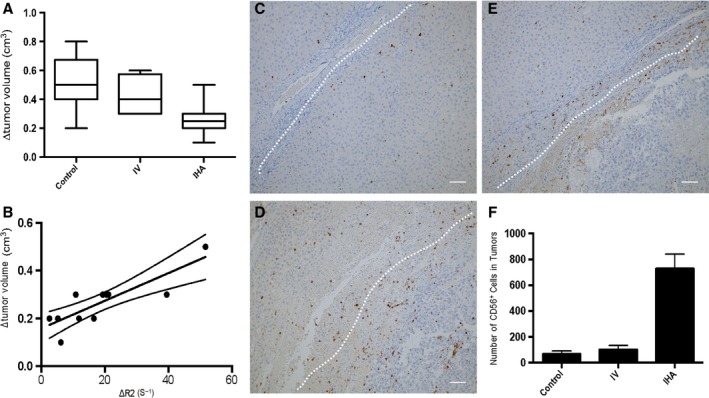
A, graph shows the changes of tumor volumes at 8 days after IHA infusion. Effective inhibition of tumor growth can be observed 8 days after IHA infusion (control group vs. IHA group, *P* < 0.001; IHA group vs. IV group, *P* = 0.001); no significant difference in therapeutic response was observed between control group and IV group 8 days after infusion (*P* = 0.196). B, graph shows the correlation between the changes of R2* measurements at 24 h postinfusion and longitudinal therapeutic response (tumor volume changes) (*R* ^2^ = 0.704, *P *<* *0.001). B–D, representative CD56 stained slices from tumors 8 days after infusion (B, control group, C, IV group, and D, IHA group). Dashed line indicates tumor border. E, the quantitative measurements of CD56‐positive NKs (IHA group vs. IV group, *P *<* *0.0001; IHA group vs. control group, *P *<* *0.0001; IV vs. control, *P *=* *0.004). B–D, scale bars = 100 *μ*m.

The efficacy of the LNK cell therapy was evident as inhibition of tumor growth in the IHA group when compared to that in control/IV animals 8 days after infusion (Δvolume: control vs. IHA, *P *<* *0.001; IV vs. IHA, *P *=* *0.001). There were no significant differences in therapeutic responses between the control group and IV group animals 8 days after infusion (*P *=* *0.196) (Fig. 6A). No significant differences were observed in Δvolume between the control and IV (*P *=* *0.363). Moreover, there was a strong correlation between tumor ∆R2* at 24 h postinfusion and Δvolume measurements in IHA group (*R* ^2^ = 0.704, *P *<* *0.001) (Fig. 6B).

Representative CD56 stained slices from tumors 8 days after infusion are shown in Figure 6C–E. The quantitative measurements of CD56‐positive LNKs are shown in Fig 6F. There were a significantly larger number of positive‐stained LNKs measured within tumors for the IHA group than for the IV systemic delivery group and control group. There were also a significantly larger number of positively stained LNKs measured in the IV group than those in the control group.

## Discussion

Our study demonstrated the following: (1) clinically applicable HPF‐labeled NKs can be tracked with MRI, (2) transcatheter IHA NK infusion offers the potential to radically augment NK cell homing efficiency to liver tumors, and (3) the change of tumor R2* value at 24 h postinfusion is an important early biomarker for prediction of longitudinal response.

NK‐ATI is a promising approach for the treatment of solid tumors including HCC 21, 22. However, conventional intravenous (IV) delivery of NKs may limit the number of cells that ultimately reach the target tumor(s) and surrounding liver tissues 17, 22, 23. Because hepatic arteries supply ~90% of the blood flow in HCC 24, the goal of transcatheter IHA delivery is to selectively infuse NKs to targeted HCC lesions for improved therapeutic efficacy. Transcatheter IHA infusion methods were described in previous studies 17, 18, 19 which used human NK cell line (NK‐92MI) with FeOlabel Texas Red (Genovis AB, Lund, Sweden) nanoparticles labeling. However, FeOlabel Texas Red nanoparticles are not approved by FDA for clini settings. This study design has a strong focus on clinical translation. All methods and materials proposed in this study are approved by the FDA for direct clinical uses, and our results are directly applicable to humans.

Furthermore, in vivo noninvasive tracking of NK cell migration to tumors will be critical in predicting early therapeutic responses 25, 26, 27, 28. We used a clinically applicable approach of combining FDA‐approved drugs to form HPF nanocomplexes for magnetic NK labeling so that NKs could be visualized by MRI 12, 29. The HPF nanocomplexes have similar biochemical properties to iron nanoparticles, which have been used previously to label cells through the iron metabolic pathway 12, 29. We optimized the labeling protocol, which showed significant uptake of HPF with minimal effect on cell viability and cytotoxicity. Our observations were in agreement with previous studies 12, 29. In our study, the doses of 50, 100, and 200 *μ*g/mL HPF were used for NK labeling, resulting in high labeling efficiency. However, iron uptake per cell was dependent upon HPF concentrations. A significant increase in cell death was found in both 100 *μ*g/mL and 200 *μ*g/mL HPF labeling groups when compared to the control group. Therefore, the dose of 50 *μ*g/mL HPH was selected for our study. The 12‐h labeling time with 50 *μ*g/mL HPf resulted in 87 ± 7% labeling efficiency, with no effect on NK cell viability and cytotoxicity. This labeling approach and 12‐h labeling period may be more conducive to clinical translation during NK‐ATI trials. This limited‐duration labeling period could potentially avoid NK function alterations and cell damage.

In vivo MRI shows extensive HPF‐labeled NKs homing to the targeted tumor shortly after transcatheter IHA NK delivery. A tumor R2* increase was observed within 24 h of NK delivery and correlated strongly with therapeutic responses (tumor growth inhibition 8 days after NK infusion). Quantification of NK measurements showed that the highest amounts of CD56‐positive NKs in tumors were present in the IHA NK group, while much fewer were present in the IV NK group with the least NKs evident in the IHA saline group.

Our study had several limitations. MR imaging of HPF‐labeled NK homing to targeted tumors was performed at only two time‐points after injection (24 h and 48 h), and the study protocol examined only one NK dose that was selected from our initial experiences. Additional studies may be valuable to determine the optimal doses and postinjection time intervals for follow‐up imaging measurements.

In summary, our study demonstrated that (1) NK cells labeled with clinically applicable approaches can be tracked with MRI, (2) transcatheter IHA NK infusion improves NK cell homing efficacy to targeted tumors, and (3) serial MRI monitoring of NK cell migration to targeted tumors may serve as an important early biomarker for prediction of longitudinal response.

## Conflicts of Interest

All authors have no potential conflicts of interest to disclose.
